# Positive effects of lignocellulose on the formation and stability of aerobic granular sludge

**DOI:** 10.3389/fmicb.2023.1254152

**Published:** 2023-08-21

**Authors:** Jie Xu, Yuan Gao, Xuejun Bi, Lin Li, Wenjuan Xiang, Shichang Liu

**Affiliations:** ^1^School of Environmental and Municipal Engineering, Qingdao University of Technology, Qingdao, China; ^2^Key Laboratory of the Three Gorges Reservoir Region’s Eco-Environment, Ministry of Education, College of Environment and Ecology, Chongqing University, Chongqing, China

**Keywords:** aerobic granular sludge, lignocellulose, extracellular polymeric substances, wastewater treatment, microbial community structure

## Abstract

**Introduction:**

Lignocellulose is one of the major components of particulate organic matter in sewage, which has a significant influence on biological wastewater treatment process. However, the effect of lignocellulose on aerobic granular sludge (AGS) system is still unknown.

**Methods:**

In this study, two reactors were operated over 5 months to investigate the effect of lignocellulose on granulation process, structure stability and pollutants removal of AGS.

**Results and discussion:**

The results indicated that lignocellulose not only promoted the secretion of tightly bound polysaccharide in extracellular polymeric substances, but also acted as skeletons within granules, thereby facilitating AGS formation, and enhancing structural strength. Lignocellulose imposed little effect on the removal efficiency of pollutants, with more than 95, 99, and 92% of COD, NH_4_^+^-N, and PO_4_^3−^-P were removed in both reactors. However, it did exhibit a noticeable influence on pollutants conversion processes. This might be due to that the presence of lignocellulose promoted the enrichment of functional microorganisms, including *Candidatus_Accumulibacter*, *Candidatus_Competibacter*, *Nitrosomonas*, and *Nitrospira*, etc. These findings might provide valuable insights into the control strategy of lignocellulose in practical AGS systems.

## 1. Introduction

Aerobic granular sludge (AGS) technology has gained widespread attention as an excellent replacement for conventional activated sludge (CAS) due to its significant advantages (Han, 2022). It has been proved that AGS could achieve excellent performance in the treatment of municipal wastewater as well as industrial wastewater from textiles (Lourenço et al., 2015), pharmaceuticals (Amorim et al., 2014), papermaking (Vashi et al., 2019), petrochemicals (Campo and Di Bella, 2019), etc. However, the application of AGS technology in practical engineering is significantly constrained by challenges such as prolonged granulation time and poor stability under unfavorable conditions (Franca et al., 2018).

The influent substrates have a significant influence on the formation and stability of AGS (Szabó, 2017; De Sousa Rollemberg et al., 2018; Adler and Holliger, 2020). For example, when glucose was applied as the sole carbon source, the cultivated AGS appeared as loose hairy granules due to the excessive proliferation of filamentous bacteria (Moy et al., 2002); when applying sodium acetate (NaAc) as the carbon source, the dominant microorganisms in granules were cocci, resulting in a more compact structure (Nancharaiah and Kiran, 2018); however, when a mixture of glucose and NaAc was applied, the surface of AGS was dominated by brevibacterium, while the interior part was dominated by cocci, exhibiting a distinct hierarchical structure (Bao et al., 2009). Lashkarizadeh et al. (2015) investigated the impact of real domestic wastewater on AGS cultivated with synthetic wastewater. The results showed that a significant granule disintegration occurred when the influent was switched from synthetic wastewater to real domestic wastewater. Although regranulation occurred after 30 d, the morphology and performance of the newly formed granules differed significantly from the initial ones. This might be due to that the complex carbon source composition in actual domestic sewage affected the microbial community and extracellular polymeric substances (EPS) characteristics in granules, thereby influencing the properties of AGS (Lemaire et al., 2008).

Particulate Organic Matter (POM) is one of the primary components of organic matter in domestic wastewater, accounting for over 60% of the organic loading (Rickert and Hunter, 1967). Studies have shown that particle size had a significant impact on the removal process of POM. Most particles larger than 100 μm could be effectively removed through sedimentation (Morgenroth et al., 2002), but smaller particles tended to enter the biological treatment system and be removed through the adsorption and entrapment by bio-aggregates (Levine et al., 1991). Currently, researches on POM in sewage treatment were mostly focused on activated sludge process, while few related studies were conducted with AGS. Cetin et al. (2018) demonstrated that high influent suspended solids (SS) content after coarse screening resulted in smaller and more stable mature granules in AGS reactors treating domestic wastewater. However, in this experiment, the specific categorization of SS was absent. Schwarzenbeck et al. (2004) and Wagner et al. (2015) investigated the impact of barley flour and starch on AGS. It was found that the hydrolysis of barley flour and starch particles adsorbed on the surface of AGS resulted in the outward extension of filamentous bacteria, leading to a decrease in granule structural stability. Nevertheless, the content of barley flour and starch in domestic wastewater is relatively low and their hydrolysis rates are relatively fast. Compared to them, lignocellulose, which constitutes 30–50% of SS in domestic wastewater, is evidently more representative (Crutchik et al., 2018). The complex structure of lignocellulose, with varied bonding interactions among cellulose, hemicellulose, and lignin, renders it a challenging substrate for enzymatic degradation (Andlar et al., 2018). Generally, the removal efficiency of lignocellulose by primary clarification is less than 50%. Therefore, a considerable portion of lignocellulose will enter the biological treatment process. Ruiken et al. (2013) and Benneouala et al. (2017) reported that the hydrolysis of lignocellulose in activated sludge took at least 10 d, and the removal of lignocellulose could effectively reduce the energy consumption of wastewater treatment plant. However, the effects of removal or biodegradation of lignocellulose on the oxygen demand, sludge production, nutrient removal and dewaterability are clear knowledge gaps. Exploring the influence of lignocellulose on the formation and properties of AGS is of great significance for the long-term stable operation of AGS system in practical engineering, but the related research has not been reported as far as we know.

In this study, toilet paper was used as the source of lignocellulose. Two AGS-SBR reactors were operated in the absence (R1) and presence (R2) of lignocellulose to investigate its effects on the granules. During the experiment, the sludge morphology, microstructure, mechanical strength, EPS, as well as the removal efficiencies of pollutants, were detected to study the physicochemical variations of the granules. High-throughput sequencing technology was employed to analyze the succession of microbial community, aiming to clarify the influence mechanism of lignocellulose on the formation and properties of AGS.

## 2. Materials and methods

### 2.1. Preparation of lignocellulose

The source of lignocellulose in this study was toilet paper produced by Hengan International Group Co., Ltd. Firstly, the toilet paper was subjected to a 3 min crushing process in a grinder. After pulverization, it was mixed with tap water in a conical flask with magnetic stirring at 1,000 rpm for 2 h. This process allowed the toilet paper to disperse evenly in tap water under shear force, effectively simulating the state of lignocellulose in real domestic wastewater.

### 2.2. Reactors operation

The cultivation of AGS was carried out using two lab-scale SBR reactors, which were composed of reactor column, inlet system, aeration system, drainage system, automatic control system, and supporting pipelines. The effective volume of the reactors was 3 L, with 7 cm internal diameter, and 80 cm effective height (H/d = 11.4). During aeration, the gas flow rate was kept at 3 L/min using glass flowmeters, corresponding to a superficial gas velocity of 1.3 cm/s. The drainage solenoid valves were installed at the midpoint of the effective height of the reactors, allowing for a volume exchange ratio of 50%. The operating cycle was kept at 3 h, and consisted of the following stages: feeding (60 min), aeration (100–111 min), settling (15–4 min), and drainage (5 min). Dissolved oxygen (DO), temperature, and pH were not controlled during operation.

### 2.3. Seeding sludge and synthetic wastewater

The seeding sludge used in this experiment was obtained from the secondary sedimentation tank of the integrated fixed-film activated sludge (IFAS) process at Tuandao Wastewater Treatment Plant in Qingdao, China. The sludge diameter was measured to be 76.9 μm, and the initial sludge concentrations in the reactors after inoculation were 3.5 g/L. To avoid external interference caused by fluctuations in actual wastewater quality, this experiment utilized synthetic wastewater as the feeding. Glucose and NaAc were applied as the mainly carbon sources with a ratio of 1:2. Ammonium chloride (NH_4_Cl) was applied as the sole nitrogen source. Phosphorus sources were provided by dipotassium phosphate (K_2_HPO_4_) and monopotassium phosphate (KH_2_PO_4_). The final concentrations of soluble COD (sCOD), NH_4_^+^-N, and PO_4_^3−^-P in the synthetic wastewater were 520 mg/L, 55 mg/L, and 9.7 mg/L, respectively. Other components in the synthetic wastewater included 20 mg/L FeSO_4_·7H_2_O, 100 mg/L CaCl_2_, 15 mg/L MgSO_4_·7H_2_O, 35 mg/L K_2_HPO_4_, 15 mg/L KH_2_PO_4_, and 1 mL/L trace elements (Vishniac and Santer, 1957). In R2, an additional 80 mg/L pretreated lignocellulose (40% of average SS concentrations, accounting for 112 mg/L of total COD) was added to the influent (Crutchik et al., 2018).

### 2.4. Analysis methods

The sludge characteristics (ML(V)SS, SVI), and water quality (COD, NH_4_^+^-N, nitrogen oxides (NO_3_^−^-N, NO_2_^−^-N), and PO_4_^3−^-P) were measured regularly according to standard methods (Eaton et al., 2005). The diameter of AGS at different periods was measured using laser particle size analyzer (Mastersizer-3000) and wet sieving method (Kishida et al., 2012). After homogenization treatment of AGS, the loosely bound EPS (LB-EPS) and tightly bound EPS (TB-EPS) were extracted using the thermal hydrolysis method (Laguna et al., 1999). The content of protein (PN) and polysaccharide (PS) in EPS was determined using the improved BCA protein assay kit from Sangon Biotech (Shanghai) Co., Ltd. and the phenol-sulfuric acid method (Gerhardt et al., 1994), respectively. The macroscopic morphology and microscopic structure of AGS were monitored using a stereomicroscope (Olympus Corporation SZX2-ILLK, Japan) and a scanning electron microscope (SEM, Carl Zeiss Gemini 300, Germany). The structural strength of the granules was qualitatively determined using ultrasonic disruption experiments, as described by Xu et al. (2019).

### 2.5. High-throughput sequencing

To characterize the microbial community, sludge samples were collected at day 0, 40, 70, and 150. The mixtures were centrifuged at 4,000 g and 4°C for 10 min, and the supernatant was discarded. Genomic DNA was extracted from samples using E.Z.N.ATM Mag-Bind Soil DNA Kit (M5635-02, OMEGA, United States) following the manufacturer’s instructions. The extracted DNA was amplified using universal primers targeting the V3-V4 region of the bacterial 16S rDNA gene (forward primer 341F and reverse primer 805R). The polymerase chain reactions (PCR) were conducted for two rounds and the products above 400 bps were purified using Agencourt AMPure XP (Beckman, United States) and quantified using the Qubit 2.0 DNA detection kit (Life, United States). The final products were sequenced on the Illumina Miseq^™^ platform by Majorbio Company (Shanghai, China). The sequencing data were analyzed using the Majorbio cloud platform.

## 3. Results and discussion

### 3.1. Formation of aerobic granules

In order to investigate the influence of lignocellulose on the formation and stability of AGS, the diameter, MLSS, morphology, and mechanical strength of the sludge were measured during operation. Based on the overall trends observed in the two reactors, the entire experimental process could be divided into four stages.

**Figure 1:**
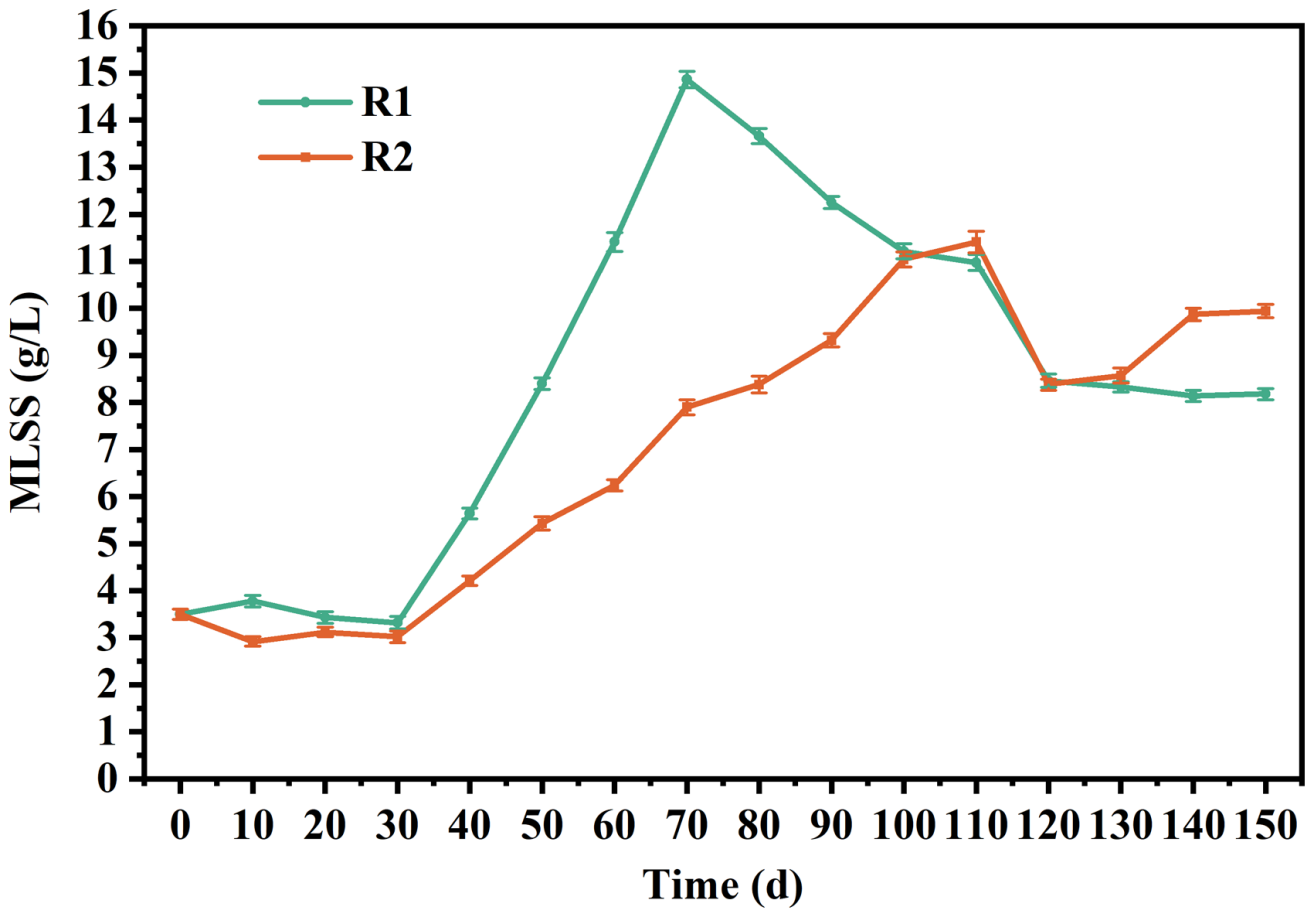
MLSS concentration in R1 and R2.

**Figure 2:**
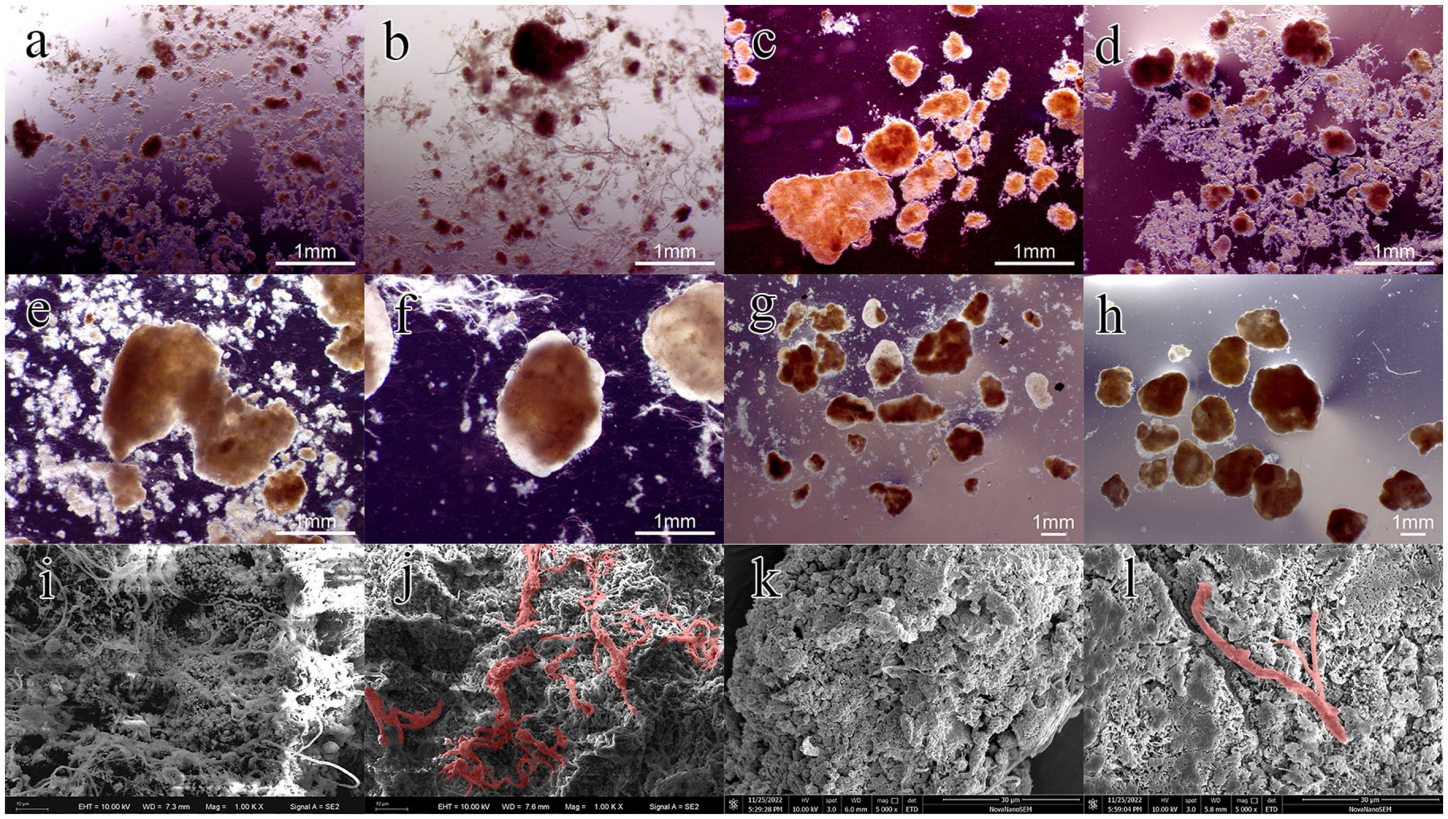
Images of granules at different stages of the granulation process: granules at day 20 (**A**, R1; **B**, R2), day 30 (**C**, R1; **D**, R2), day 100 (**E**, R1; **F**, R2) and day 150 (**G**, R1; **H**, R2); SEM of AGS at day 30 (**I**, R1; **J**, R2) and day 150 (**K**, R1; **L**, R2).

The first stage was from day 1 to day 30. During this stage, the settling time was gradually reduced from 15 to 4 min within 15 d, providing the necessary selection pressure for granulation. It should be pointed out that most lignocellulose was trapped by the sludge layer during the influent stage, and its penetration depth was less than 10 cm. The mixing and contact between lignocellulose and sludge mainly occurred during aeration. As shown in Figure 1, the concentration of MLSS in R2 decreased to 2.93 g/L within 10 d and remained relatively stable thereafter. In contrast, it initially increased to 3.78 g/L and then gradually decreased to 3.32 g/L in R1. This might be attributed to the poor settling ability of lignocellulose, which entrapped and retained flocs during settling, leading to increased screening intensity. It should be noted that small granules appeared in R2 on day 15, which was about 5 d earlier than R1. Figures 2A,B are the stereo microscope images of the sludge on day 20. It can be seen that lignocellulose was wrapped around granules, like filamentous bacteria, resulting in a larger granule size and less free flocs in R2. Most flocs were gathered around lignocellulose, indicating that lignocellulose might promote the formation of AGS by acting as skeletons for sludge aggregating. However, starting from day 20, a rapid increase in the proportion of granular sludge in R1 occurred, and its average diameter reached 311 μm by the 30th day, which was higher than the 217 μm observed in R2. As shown in Supplementary Figure A1, the size distribution in R1 was more concentrated, and the proportion of granular sludge (diameter > 200 μm) reaching 74.2%, while the proportion of granular sludge in R2 was only 43.92%. Figures 2C,D show that granules in R1 at this point had a loose and irregular morphology, while granules in R2 exhibited more compact structure. However, due to the presence of the extended lignocellulose on the AGS surface, a significant amount of flocs were trapped around granules, which might be the main reason for the lower proportion of AGS in R2. SEM images reveal that the granules in R1 (Figure 2I) were formed on the basis of the skeleton structure of filamentous bacteria. However, the granules in R2 (Figure 2J) were primarily supported by lignocellulose, and there was no significant outward expansion of filamentous bacteria. This phenomenon contrasted with the results reported by Wagner et al. (2015) and Schwarzenbeck et al. (2004), which might be attributed to the relatively rapid hydrolysis rate of barley flour and starch by microorganisms, resulting in an increased substrate gradient in AGS, thereby inducing the outward proliferation of filamentous bacteria. Nevertheless, the structure of lignocellulose is intricate, and the hydrolysis process requires the cooperation of multiple microorganisms (Berlin et al., 2010). Therefore, the hydrolysis rate was much slow and mainly occurred inside granules, which impeded the outwards proliferation of filamentous bacteria.

Phase II was from day 30–70. During this period, the biomass in both reactors increased rapidly, reaching 14.86 and 7.9 g/L on day 70, respectively. The average growth rate of MLSS in R1 was about 0.29 g/L/d, which was 2.42 times of that in R2. The reason for this result might be that AGS had a low interception efficiency of lignocellulose, which led to the discharge of the sludge with poor sedimentation performance along with lignocellulose, resulting in higher SS levels in the effluent of R2. Starting from day 70 (Phase III), disintegration of granules in R1 occurred. A part of fragmented granules was discharged with the drainage, resulting in a rapid decrease in MLSS of R1, reaching 11.21 g/L on day 110. As shown in Figure 2E, hollow structure and excessive proliferation of filamentous bacteria were observed in the granules, which might be the main reasons for granule disintegration (Adav et al., 2008). However, this phenomenon did not appear in R2, and the MLSS growth rate was close to that of phase II (Figure 2F), indicating that the presence of lignocellulose could improve the stability of AGS.

From day 110, the sludge discharge strategy was implemented to maintain a sludge retention time (SRT) of 30 d, which stabilized the MLSS concentrations in the two reactors at 8.2 and 9.9 g/L, respectively. The distribution of granule size was measured at day 120 and 150. As shown in Supplementary Figure A2, the average diameter of AGS in R1 decreased from 1.47 to 1.08 mm, indicating an ongoing granule disintegration. However, the size distribution in R1 became more concentrated by day 150, with the AGS percentage increasing from 74.77 to 97.57%, revealing a successful regranulation. As shown in Figures 2G,H, the granules in both reactors were irregular spheres, but the granules in R2 exhibited a higher degree of integrity and roundness. SEM images reveal that the AGS in R1 (Figure 2K) were mainly composed of cocci. In R2 (Figure 2L), the microbial species were more abundant, while bacilli, brevibacterium and cocci can all be found on the surface of granules. It should be noted that the proportion of lignocellulose in R2 was significantly reduced compared to phase I, which is consistent with the low retention efficiency of lignocellulose by granular sludge mentioned above.

**Figure 3:**
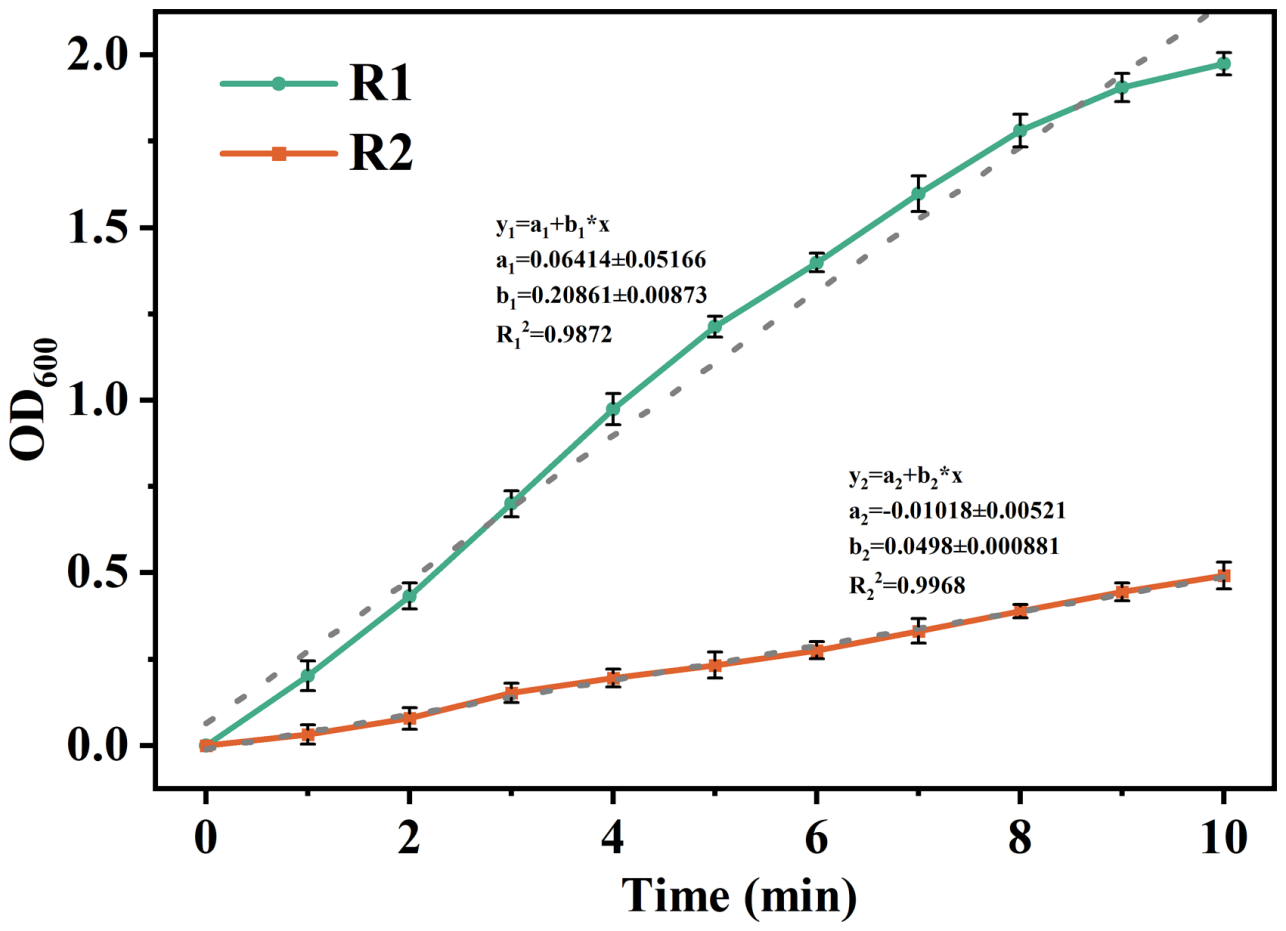
Variation of OD_600_ in supernatant of mature granules under 60 W ultrasound.

To further evaluate the enhancement of lignocellulose on the structural stability of AGS, the mature granules were subjected to ultrasonic fragmentation at 60 W. As shown in Figure 3, the increasing rate of OD_600_ values in the supernatant represents the granule crushing rate. It can be found that the OD_600_ values in both reactors increased continuously after the beginning of ultrasound treatment. The linear fitting showed that the granule crushing process in R2 was more gentle, and the crushing rate of AGS-R2 was only 23.8% of that of AGS-R1, indicating that the lignocellulose skeleton could effectively enhance the stability of AGS by providing a more uniform inter-granule compactness.

### 3.2. Pollutants removal performance

**Figure 4:**
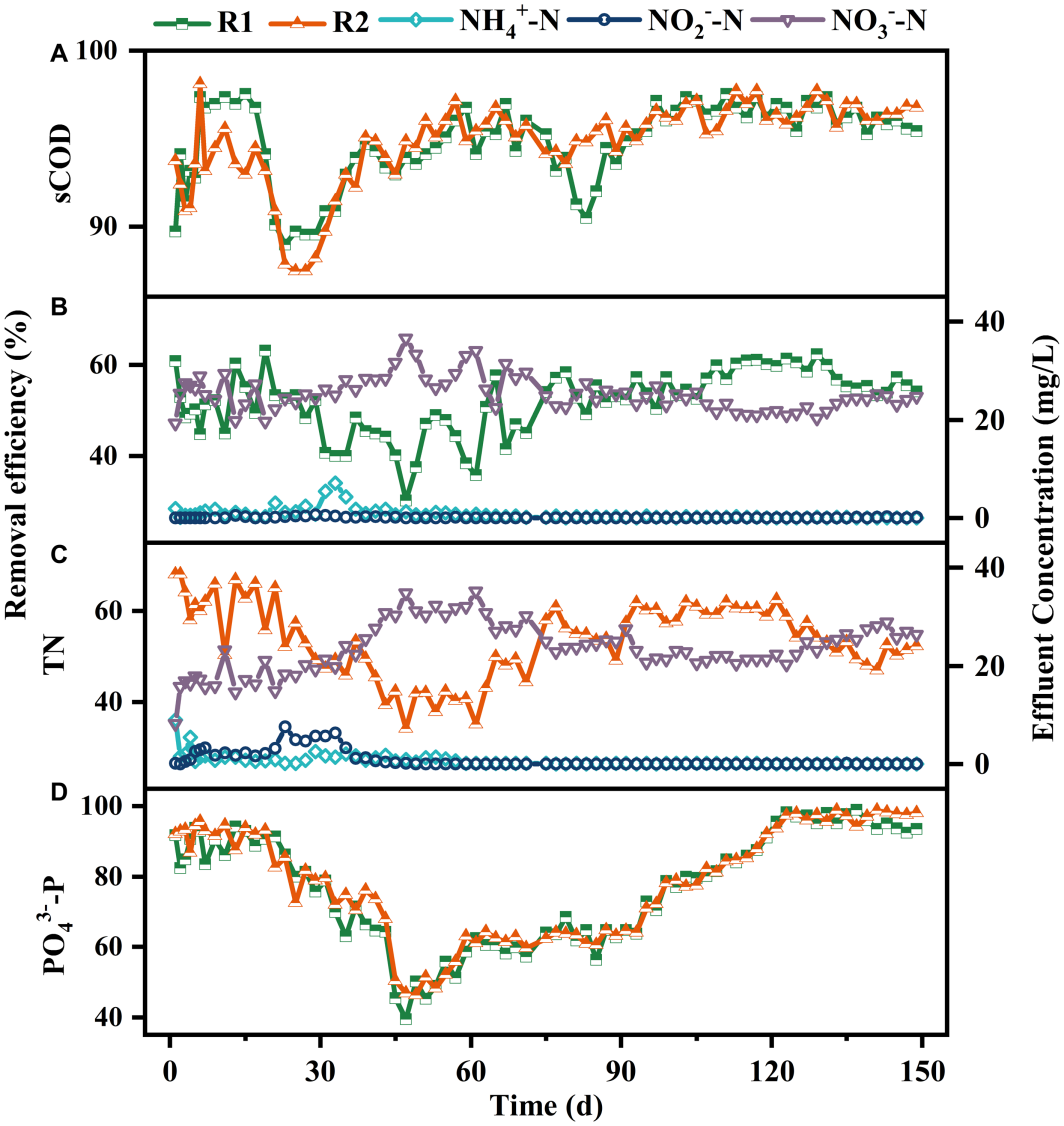
Removal performance on sCOD **(A)**, TN-R1 **(B)**, TN-R2 **(C)**, PO_4_^3−^-P **(D)** and effluent concentrations of NH_4_^+^-N, NO_2_^−^-N and NO_3_^−^-N in R1 **(B)** and R2 **(C)**.

The pollutants removal performance in both reactors was analyzed throughout the experiment. As shown in Figure 4, the variations of pollutants removal efficiencies in both reactors were similar generally. As shown in Figure 4A, after a short adaptation period, the sCOD removal rate in R1 increased to above 97%, which was approximately 3% higher than R2. From day 19, there was a significant decrease in sCOD removal efficiency in both reactors, reaching 88.96 and 87.44% by day 23. This might be attributed to the loss of sludge and increased sieving strength caused by the presence of lignocellulose. Subsequently, as the granules grew, the sCOD removal efficiency in R2 gradually increased to over 95% and remained stable thereafter. However, a significant decrease of sCOD removal rate was observed in R1 on day 81, which might be due to the sludge loss caused by granule disintegration. As the broken sludge gradually adapted to the operating environment, its sCOD removal efficiency recovered within 6 d.

In terms of nitrogen removal, the differences also appeared in the first stage. As shown in Figures 4B,C, both reactors achieved over 98% NH_4_^+^-N removal efficiency within 15 d, but NO_2_^−^-N was significantly accumulated in R2, which reached 5.74 mg/L on day 29. In contrast, the effluent NO_3_^−^-N concentration was much higher in R1, indicating a higher nitrite oxidation rate. The different loss rate of nitrite oxidation bacteria (NOB) during sludge selecting might be the main reason for this phenomenon (Winkler et al., 2012). As the granules matured, the TN removal efficiencies in R1 and R2 were basically maintained at approximately 50% until the end of operation.

In the enhanced phosphorus removal system, the removal of phosphorus is primarily achieved by the excessive uptake by polyphosphate-accumulating organisms (PAOs), and SRT is one of the main influencing factors for phosphorus removal efficiency (Campo et al., 2020). Therefore, the significantly different sludge quantity and effluent SS concentrations between the two reactors were supposed to cause different PO_4_^3−^-P removal performance. Surprisingly, the two reactors displayed similar PO_4_^3−^-P removal performance throughout the experiment. As shown in Figure 4D, the PO_4_^3−^-P removal efficiency in both reactors remained above 80% during the first 23 d. After that, a rapid decrease occurred and the removal efficiency reduced to 39.48 and 46.8% within 20 d. Although there was a slight increase in the following 48 d, the PO_4_^3−^-P removal efficiency remained below 65%, even during the granule disintegration phase in R1. Temperature fluctuations might be the primary factor contributing to this result. As described by Ab Halim et al. (2016), high temperature could inhibit the uptake of carbon sources by PAOs, resulting in a disadvantage position of PAOs in the competition with glycogen-accumulating organisms (GAOs). Since the operating temperature was not controlled during the experiment, the temperature inside the reactors increased to above 25°C from day 27, and the highest temperature could reach 35°C (data not shown). As the environmental temperature decreased from day 95, the removal efficiency of PO_4_^3−^-P increased to around 80% gradually. Subsequently, with the implementation of the sludge discharge strategy, the PO_4_^3−^-P removal efficiency increased to over 92 and 97% within 10 d, respectively, and remained stable thereafter.

The pollutants conversion processes in typical cycle were detected on day 150 to further identify the effect of lignocellulose on AGS. As shown in Supplementary Figure A3, significant differences appeared in terms of N and PO_4_^3−^-P removal. After feeding, the NO_3_^−^-N concentration in R1 decreased by 23.54% except for influent dilution, which was 13.81% higher than that in R2, implying a higher traditional denitrification efficiency. During the aeration phase, the concentration of NH_4_^+^-N in R2 decreased to below 5 mg/L within 60 min, corresponding to an ammonia oxidation rate of 1.87 mgNH_4_^+^-N/gMLSS/h, which was 1.56 times higher than that of R1. It is worth noting that during the first 20 min of aeration, the increase rate of NO_3_^−^-N in R2 was much lower than that in R1, indicating that R2 had a higher simultaneous nitrification–denitrification efficiency. In terms of phosphorus removal, it can be observed that the phosphorus release and accumulation rates in R2 were 2.28 and 3.20 mgP/gMLSS, respectively, which were 2.57 and 3.14 times higher than that in R1, revealing that the presence of lignocellulose could effectively enhance the activity of PAOs.

### 3.3. Characteristics of EPS

**Figure 5:**
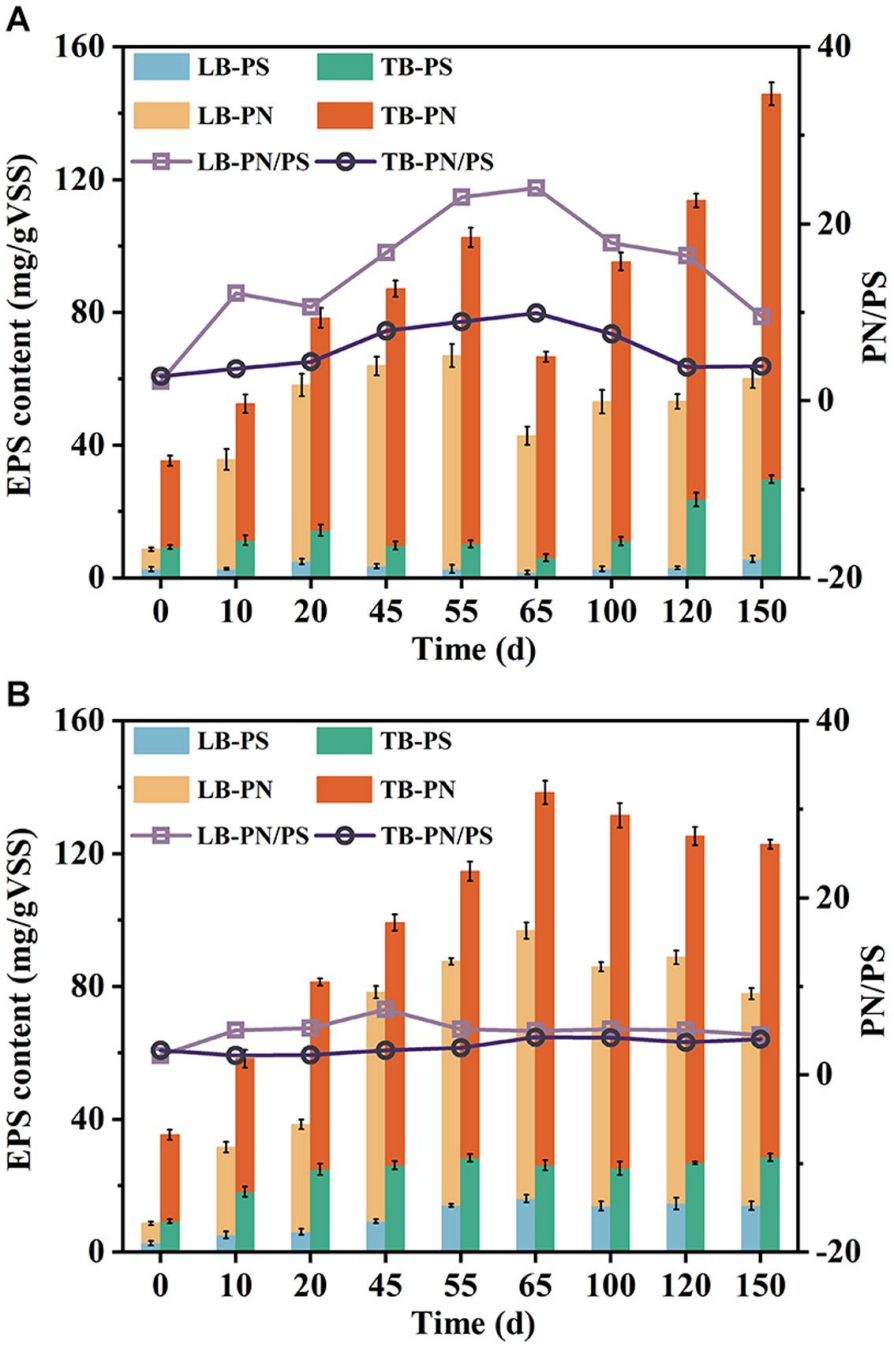
Variation of PN and PS content of LB-EPS and TB-EPS during the operation in R1 **(A)** and R2 **(B)**.

EPS are crucial components of AGS, playing a significant role in microbial aggregation, granule formation, and structural stability maintenance (McSwain et al., 2005). As shown in Figures 5A,B, the EPS content exhibited an upward trend in both systems during the early stages of operation. R1 showed a faster increase rate in TB-PN, while R2 exhibited a faster increase rate in TB-PS. By the 55th day, the TB-PS content in R2 had reached 28.38 mg/gVSS, which was 7.15 times higher than that in R1. There might be three hypotheses for this phenomenon. Firstly, the toilet paper used here contained a certain amount of cellulose, which might be extracted and detected as PS (Ruiken et al., 2013). Secondly, the lignocellulose present in the system could be hydrolyzed by microorganisms, releasing cellulose and hemicellulose from its structure (Chen et al., 2017). Lastly, the presence of lignocellulose stimulated microorganisms to secrete more PS. Additional research is required to further confirm the specific causes for this phenomenon. It should be noticed that the TB-PS in R1 showed an initial upward trend followed by a decline during the first two stages. At the end of phase II (day 65), a significant decrease in EPS content occurred, particularly in TB-PN and TB-PS, which reduced by 34.41 and 40.6%, respectively. By comparison, EPS in R2 continued to increase during this stage and reached a stable level after day 65. Studies have shown that PN and PS played different roles in the structure of AGS (McSwain et al., 2005). PS, especially β-PS, served as the skeleton for maintaining the structural stability of AGS, while PN acted as the fillings (Adav et al., 2010). In this study, the increase of PN content in R1 was consistent with the formation and amplification process of granular sludge, while the decrease of TB-PS content had indicated a decline in its supporting capacity. Simultaneously, the outward extension of filamentous bacteria resulted in increased granule irregularity, ultimately leading to the disintegration of AGS under the action of hydraulic shear force. In the later stages of the experiment, the PN and PS content in R1 gradually recovered, with a comparable TB-PS and 1.23 times higher TB-PN content than that of R2, which was consistent with its regranulation process.

The increase of PN/PS ratio has always been regarded as an important indicator of sludge granulation (Franca et al., 2018). In this study, the LB-PN/PS and TB-PN/PS ratios in R1 and R2 exhibited an increasing trend during granulation, but decreasing in mature granules. Furthermore, despite the lower PN/PS values in AGS-R2, it exhibited significantly better performance in granule formation and higher structural stability, indicating that the content of EPS, especially TB-PS, played a more crucial role in AGS than PN/PS ratio.

### 3.4. Characteristics of microbial community

High-throughput sequencing technology was employed to investigate the differences in microbial community structure between R1 and R2. Sludge samples were collected at different stages, including Day 0 (SEED), Day 40 (R1-1 & R2-1), Day 70 (R1-2 & R2-2), and Day 150 (R1-3 & R2-3). As shown in Supplementary Table A1, the highest community richness and diversity were observed in SEED. As the reactors operated, the richness in R1 and R2 decreased continuously, which was approximately half of that in SEED at the end of the experiment, indicating a significant microbial selection effect during granulation. Besides, the values of Shannon, Ace and Chao index in R2-3 were higher compared to R1-3, indicating that the addition of lignocellulose could improve the microbial community richness and diversity in mature AGS.

**Figure 6:**
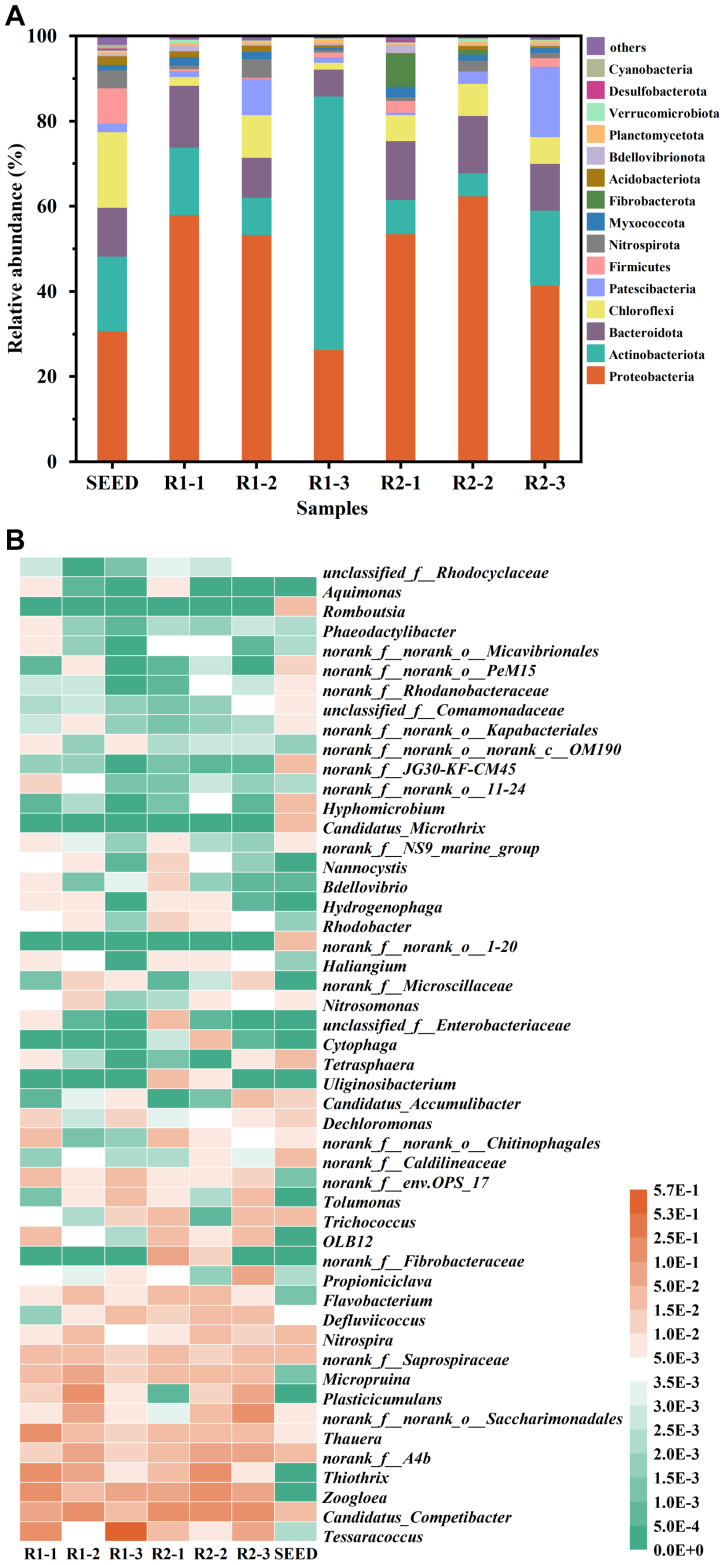
Relative abundance of the main phylum **(A)** and heat-map of the identified genus **(B)** of the seed sludge and AGS samples in R1and R2 at day 40, 70, and 150.

Figure 6A shows the relative abundance of main phyla in different samples. It can be seen that the predominant phyla (relative abundance >5%) were Proteobacteria, Actinobacteriota, Bacteroidota, Chloroflexi, Patescibacteria, and Fibrobacterota. Both abundances of Proteobacteria and Bacteroidota increased initially and then decreased during granulation. Interestingly, Actinobacteriota showed an opposite trend, and its relative abundance in R1-3 was 59.5%, which was in an absolute dominant position. Chloroflexi is usually filamentary and mostly exists in EPS with PS as the substrate for metabolism (Kragelund et al., 2007). In this study, the relative abundance of Chloroflexi in R2 remained relatively stable around 6.5%, while it was negatively related to the TB-PS content in R1, suggesting that the excessive growth of Chloroflexi could be one reason for the instability of AGS-R1. It is worth mentioning that the relative abundance of Fibrobacterota in R2 increased from 0.03 to 7.6% during the first 40 d, but gradually decreased as the granules matured, while it was lower than the detection limit in R1. The study has shown that Fibrobacterota has the function of hydrolyzing cellulose (Xu et al., 2021). The enrichment of this phylum confirmed that the cellulose components in toilet paper could be intercepted and hydrolyzed by floc sludge. With the sludge granulation, the retention efficiency of AGS to lignocellulose reduced, therefore Fibrobacterota gradually lost its dominance position.

To further investigate the impact of lignocellulose on the microbial community succession, heat map at genus level is analyzed. As shown in Figure 6B, the dominant genera in SEED were *norank_f__Caldilineaceae*, *Nitrospira*, *Candidatus_Competibacter*, *norank_f__Caldilineaceae*, *norank_f__Saprospiraceae*, and *norank_f__A4b*. By the 40th day, the dominant bacterial genera in R1 turned to be *Thauera* (15.15%), *Thiothrix* (13%), *Tessaracoccus* (11.66%), *Zoogloea* (10.11%), and *Candidatus_Competibacter* (5.81%). However, in R2, the dominant genera were *Candidatus_Competibacter* (16.44%), *Zoogloea* (9.56%), and *norank_f__Fibrobacteraceae* (7.61%). *Thauera* is a traditional denitrifying bacteria, which can perform short-cut denitrification by reducing NO_3_^−^-N to NO_2_^−^-N under limited carbon source condition (Tao et al., 2023). The enrichment of *Thauera* in R1 might be related to the higher concentration of NO_3_^−^-N. Due to the sludge loss, the NO_2_^−^-N oxidation rate in R2 was insufficient, which could not provide sufficient NO_3_^−^-N for *Thauera*, resulting in its relatively low abundance (3.76%). *Thiothrix* is a typical filamentous bacterium (De Graaff et al., 2020), and *Zoogloea* has the ability to secrete a large amount of EPS (Xu et al., 2019). The enrichment of these two genera provided the basis for the rapid growth of AGS in R1, but the sludge expansion caused by the excessive proliferation of *Thiothrix* posed a risk to granule integrity. In R2, the relative abundance of *Zoogloea* was slightly lower than that of R1, which was inconsistent with its higher EPS content. This could be explained by the higher relative abundance of *Candidatus_Competibacter* in R2, which has been reported to be able to promote the secretion of PS-EPS, thus playing an important role in maintaining the stability of AGS (Song et al., 2022).

The microbial community structure of AGS at day 70 was drastically different from that of day 40. In R1, the relative abundances of *Thiothrix*, *Tessaracoccus*, and *Zoogloea* decreased to 6.26, 0.44, and 3.68%, while the relative abundances of *Candidatus_Competibacter*, *Plasticicumulans*, *norank_f__A4b*, *norank_f__norank_o__Saccharimonadales*, and *Micropruina* increased to 18.79, 10.87, 9.02, 7.61, and 6.52%, respectively. The decrease in relative abundance of *Zoogloea*, along with the increased relative abundances of *Plasticicumulans*, *norank_f__A4b*, and *norank_f__norank_o__Saccharimonadales*, which were known for their ability to hydrolyze EPS (Zhang et al., 2022; Jiang et al., 2023; Wang et al., 2023), collectively contributed to the reduction in EPS content during this stage. Interestingly, the relative abundance of *Thiothrix* increased to 19.25% in R2-2, however, no excessive filamentous bacteria were observed on the surface of granules in SEM images. This could be due to the fact that the hydrolysis of lignocellulose mainly occurred inside the granules, so filamentous bacteria tended to grow inward rather than outward expansion. The relative abundance of *Candidatus_Competibacter* and *Micropruina* in R2 showed a similar trend as R1, which increased to 17.57 and 3.67%, respectively. As the same with *Candidatus_Competibacter*, *Micropruina* also belongs to GAO (Shintani et al., 2000). As mentioned above, high temperature could inhibit the activity of PAOs, thereby providing GAOs a competitive advantage (Ab Halim et al., 2016), which was consistent with the low removal efficiency of PO_4_^3−^-P observed in both reactors at this stage.

It is noteworthy that *Tessaracoccus* was greatly enriched in R1-3, with a relative abundance of 57.15%. For a long time, *Tessaracoccus* has been regarded as a fermentative GAO (fGAO), but according to the study of Elahinik et al. (2022), it has been found that although *Tessaracoccus* lacks PHA synthase (phaC), it possesses some enzymes involved in poly-P metabolism, and the presence of polyphosphate kinase (ppk) suggests that *Tessaracoccus* might be capable of accumulating poly-P, but unable to utilize it for energy generation due to the absence of AMP phosphotransferase (pap). Taking into account the relatively low abundance of other PAOs (*Flavobacterium*, *Candidatus_Accumulibacter*) in R1, which were below 1%, and its lower activity in anaerobic phosphorus release and aerobic phosphorus accumulation, it can be inferred that *Tessaracoccus* is indeed involved in the phosphorus removal process, with a lower phosphorus accumulation rate compared to traditional PAOs. In R2, the combined relative abundance of *Flavobacterium* and *Candidatus_Accumulibacter* was 3.36%, which was 2.55 times higher than that in R1, resulting in a higher phosphorus conversion rate. Besides, the relative abundance of *Nitrosomonas* (AOB) and *Nitrospira* (NOB) in R2 was also 2.78 and 2.54 times higher, explaining its high ammonia oxidation and nitrite oxidation rates. It should be pointed out that the relative abundance of *norank_f__norank_o__Saccharimonadales* and *Plasticicumulans* in R2-3 increased to 15.22 and 6.13%, respectively, consistent with the decrease in EPS-PN content. Simultaneously, the relative abundance of *Thiothrix* decreased to <1%. Even so, the granules in R2 still maintained high mechanical strength, further confirming that lignocellulose could increase the structure stability of AGS.

## 4. Conclusion

This study investigated the influence of lignocellulose on the formation process and physicochemical characteristics of AGS. The results demonstrated that lignocellulose could increase the content of TB-PS in EPS. Additionally, it could act as skeletons during flocs aggregating, thus facilitating the formation of AGS with higher structural stability. Furthermore, lignocellulose could also regulate the microbial community structure of AGS, promote the enrichment of functional microorganisms such as DPAOs, AOB, and NOB, thus improving the conversion rates of pollutants. Therefore, proper control of the pretreatment process to ensure that more lignocellulose could enter the bioreactor will be an effective approach to enhance the practical application of AGS technology.

## Data availability statement

The original contributions presented in the study are included in the article/Supplementary material, further inquiries can be directed to the corresponding author.

## Author contributions

JX: Formal analysis, Funding acquisition, Methodology, Resources, Writing – review & editing, Conceptualization, Investigation. YG: Formal analysis, Investigation, Software, Visualization, Writing – original draft. XB: Formal analysis, Funding acquisition, Project administration, Supervision, Writing – review & editing. LL: Conceptualization, Project administration, Supervision, Writing – review & editing. WX: Investigation, Methodology, Visualization, Writing – review & editing. SL: Data curation, Methodology, Visualization, Writing – review & editing.

## Funding

This research was supported financially by National Natural Science Foundation of China (No. 52200061) and Natural Science Foundation of Shandong Province (No. ZR2021QE274).

## Conflict of interest

The authors declare that the research was conducted in the absence of any commercial or financial relationships that could be construed as a potential conflict of interest.

## Publisher’s note

All claims expressed in this article are solely those of the authors and do not necessarily represent those of their affiliated organizations, or those of the publisher, the editors and the reviewers. Any product that may be evaluated in this article, or claim that may be made by its manufacturer, is not guaranteed or endorsed by the publisher.
