# Supplementary material for: Positive effects of lignocellulose on the formation and stability of aerobic granular sludge
Source: Front Microbiol. 2023 Aug 21;14:1254152. doi: 10.3389/fmicb.2023.1254152 (PMC10475587; doi:10.3389/fmicb.2023.1254152)
Supplement: Supplementary file 1 [file Table_1.docx]

**Table A1** Bacterial community richness and diversity indices in different samples.

| Sample | Shannon | Simpson | Ace | Chao | Coverage |
| --- | --- | --- | --- | --- | --- |
| SEED | 5.5 | 0.01 | 954 | 949 | 0.997 |
| R1-1 | 4.04 | 0.05 | 665 | 689 | 0.996 |
| R1-2 | 4.07 | 0.04 | 689 | 678 | 0.996 |
| R1-3 | 2.57 | 0.33 | 534 | 544 | 0.996 |
| R2-1 | 4.39 | 0.03 | 644 | 683 | 0.996 |
| R2-2 | 3.91 | 0.06 | 632 | 632 | 0.996 |
| R2-3 | 4.12 | 0.04 | 590 | 564 | 0.996 |
