# Supplementary figures and images for: Positive effects of lignocellulose on the formation and stability of aerobic granular sludge
Source: Front Microbiol. 2023 Aug 21;14:1254152. doi: 10.3389/fmicb.2023.1254152 (PMC10475587; doi:10.3389/fmicb.2023.1254152)

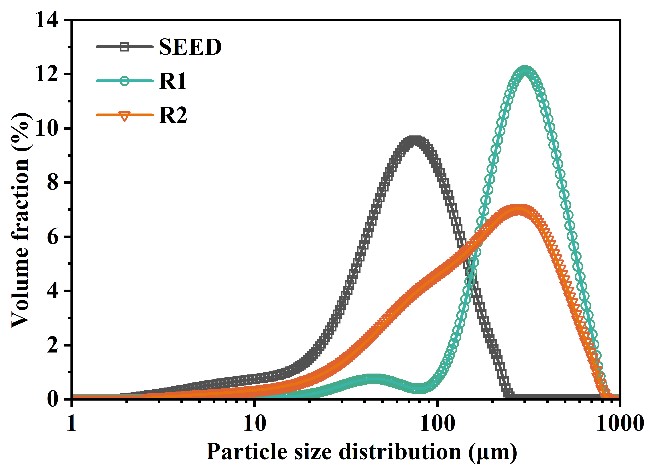

Supplement: Supplementary file 2 [file Image_1.jpg]

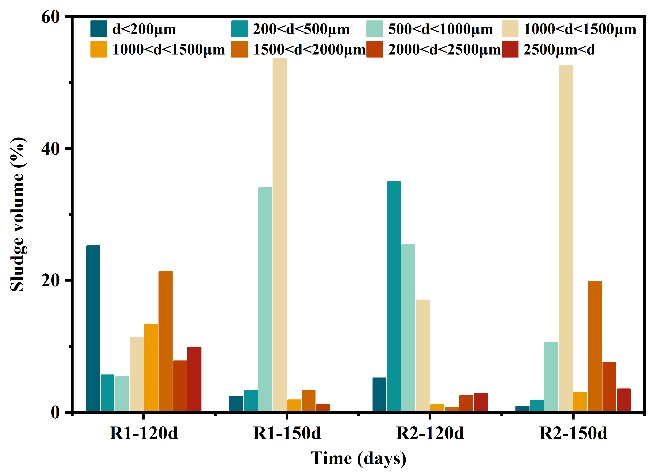

Supplement: Supplementary file 3 [file Image_2.jpg]

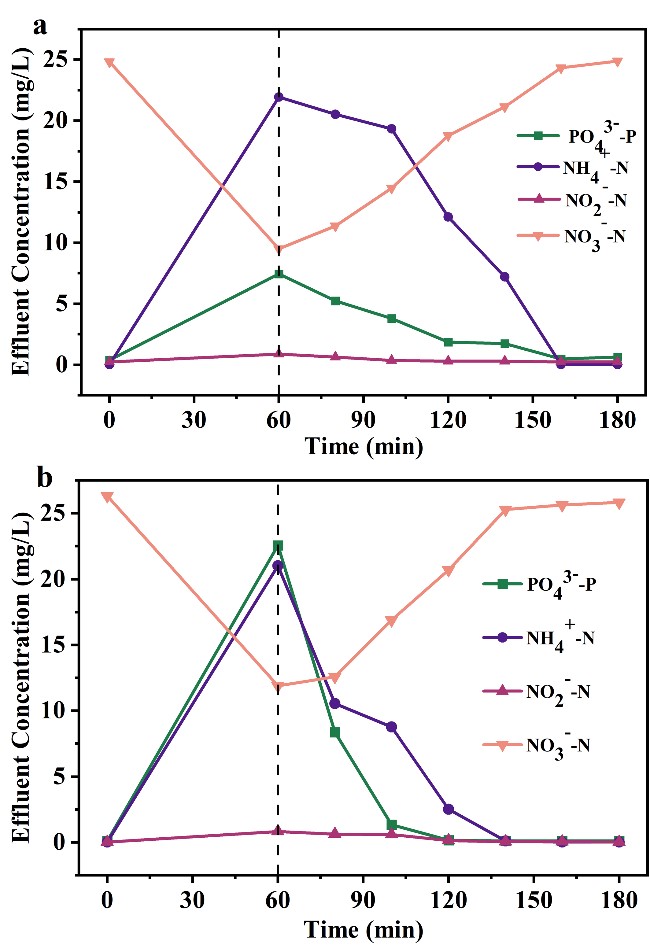

Supplement: Supplementary file 4 [file Image_3.jpg]
